# Characteristics and Diagnostic Accuracy of Retained Products of Conception After Delivery vs. Abortion: A Retrospective Cohort Study

**DOI:** 10.7759/cureus.80380

**Published:** 2025-03-11

**Authors:** Avishalom Sharon, Alex Kalendaryov, Inshirah Sgayer, Susana Mustafa Mikhail, Lior Lowenstein, Ala Aiob

**Affiliations:** 1 Obstetrics and Gynecology, Faculty of Medicine, Bar Ilan University, Safed, ISR; 2 General Medicine, Faculty of Medicine, Bar Ilan University, Safed, ISR; 3 Obstetrics and Gynecology, Galilee Medical Center, Nahariya, ISR

**Keywords:** diagnostic accuracy, hysteroscopy, post-abortion, post-delivery, residual placenta, sonographic features

## Abstract

Objective: We aimed to evaluate and compare the characteristics, clinical presentations, sonographic features, and hysteroscopic complications of retained products of conception (RPOC) after delivery versus abortion and to compare the accuracy of diagnosing RPOC in these two conditions.

Methodology: This retrospective cohort study included 226 women aged 17-50 years who underwent hysteroscopy for suspected RPOC following spontaneous or induced abortion or delivery between 2018 and 2021. The diagnosis was based on a routine ultrasound, with a hysteroscopy conducted within one week. Collected data encompassed demographics, obstetric history, sonographic findings, clinical features, operative details, and pathology reports.

Results: Of the 226 women included, 65 (28.8%) had RPOC post-delivery and 161 (71.2%) post-abortion. While age and parity did not differ significantly between the groups, gravidity was higher, and previous abortions were more common among the women post-abortion. Symptomatic presentation differed; bleeding and pain were more common post-delivery, and asymptomatic presentation was more frequent post-abortion. Sonographically, Doppler flow was more prevalent post-abortion. The optimal endometrial thickness cutoffs for predicting RPOC post-abortion and post-delivery were 1.55 and 1.49 cm, respectively.

Conclusions: Our study found significant differences in clinical and sonographic characteristics between women with RPOC after abortion versus delivery, emphasizing the importance of tailored diagnostic and management strategies. Although detection rates and sonographic findings varied, routine sonographic assessment is crucial, especially post-abortion. Endometrial thickness proved a reliable predictor for RPOC in both groups.

## Introduction

The retention of products following pregnancy-related events, such as normal deliveries, cesarean sections, spontaneous miscarriages, or intentional terminations, is a common clinical scenario encountered by gynecologists [1]. The reported incidence of this condition, known as retained products of conception (RPOC), varies widely depending on the specific pregnancy outcome and the approach used for uterine evacuation [2-4]. Studies have suggested an incidence of around 1% following full-term deliveries and a range of 0.4%-3.8% after early pregnancy losses [2]. Interestingly, the rates appear to be higher when medical management is employed for uterine evacuation, with one retrospective analysis reporting an incidence of 13% after vacuum aspiration and 29.4% after medical management [5].

The diagnosis of RPOC can be quite challenging for clinicians. Typically, the suspicion arises based on clinical symptoms, such as persistent or recurrent vaginal bleeding in the postpartum or post-abortion period [5]. Imaging modalities, including ultrasonography, color Doppler, and hysteroscopy, are then utilized to confirm the presence of residual intrauterine contents [5]. However, it is important to note that approximately 30% of women diagnosed with RPOC are asymptomatic and are often incidentally identified during routine pelvic ultrasounds after delivery or pregnancy termination [6]. Furthermore, while ultrasonography is a widely available and non-invasive diagnostic tool, its specificity for RPOC is relatively low, and the addition of color Doppler has not significantly improved its diagnostic accuracy [5].

Notably, among women with sonographic indicators of RPOC - for instance, an endometrial echogenic mass or an endometrial thickness greater than 10 mm accompanied by vascular flow - only 62% were ultimately confirmed to have RPOC. Conversely, nearly 40% of the women in that study who were suspected of having RPOC did not demonstrate any residual tissue on hysteroscopic examination and thus underwent unnecessary interventions [7]. These findings highlight the limitations of relying solely on clinical presentation and imaging characteristics for the diagnosis of RPOC, as similar sonographic and clinical features can be observed in normal postpartum and post-abortion conditions [5,8-10]. There is insufficient data about the differences in clinical presentation, sonographic features, and diagnosis accuracy between RPOC after delivery and abortions and regarding the complications of hysteroscopic intervention. Considering the gaps in knowledge, we aimed to evaluate and compare the characteristics, clinical presentations, sonographic features, and complications of the hysteroscopic intervention in RPOC after delivery vs abortion and to compare the sensitivity of diagnosing RPOC.

## Materials and methods

Before data collection, this study was approved by the Institutional Review Board (Helsinki Committee) of Galilee Medical Center and the Israeli Ministry of Health (authorization number 0042-22-NHR, June 12, 2023). The study cohort comprised women aged 17-50 years who underwent hysteroscopic evaluation for suspected retained RPOC following spontaneous or induced abortion or any delivery exhibiting risk factors for RPOC between 2018 and 2021.

Women underwent transvaginal sonography (TVS) and clinical evaluation if they presented risk factors for RPOC, such as a history of manual uterine exploration, abnormal placental appearance post-delivery, postpartum hemorrhage, placental adherence, bleeding following spontaneous or induced abortion, or previous uterine medical evacuation.

RPOC was suspected in women presenting with clinical symptoms such as vaginal bleeding, abdominal pain, or fever, along with abnormal intrauterine ultrasound findings. These findings included the presence of an endometrial mass, heterogeneous echogenic material within the endometrial cavity, increased endometrial thickness, or increased vascularity on Doppler ultrasound. Additionally, we included women who underwent hysteroscopy for suspected RPOC, regardless of whether a pathological specimen was sent for analysis or if no RPOC was observed during the hysteroscopic procedure. Exclusion criteria were the absence of documented ultrasound, hysteroscopy, or pathology reports.

Following departmental protocol, a routine TVS examination was performed 4-10 weeks after delivery or following any spontaneous or induced abortion in patients with the aforementioned risk factors or clinical symptoms of RPOC. These examinations were conducted by specialists using transvaginal probes, with both gray-scale and Doppler imaging modalities. The sonographic reports documented endometrial thickness, hyperechogenic findings, and the presence of Doppler flow - indicative of a highly vascularized lesion within the uterine cavity. The decision to proceed with hysteroscopic intervention was based on a combination of sonographic findings, clinical presentation, and identified risk factors.

A hysteroscopy was performed within one week of the suspected RPOC. An experienced gynecologist, defined as a board-certified specialist in obstetrics and gynecology with a minimum of five years of clinical experience, carefully described the uterine cavity findings, including the size and characteristics of any lesions, and noted whether RPOC was suspected. A positive histopathological result confirmed the diagnosis of RPOC. Data collected for analysis included demographic information, obstetrical history, details of the recent delivery or abortion, sonographic findings, clinical features, operative procedures, complications (e.g., uterine perforation, bleeding, or fever), and pathology reports. The histopathological diagnosis was based on chorionic villi, trophoblastic tissue, decidual remnants, or endometrial breakdown products, consistent with retained products of conception.

## Results

Our study included 226 women. Based on the source of the RPOC, they were classified as after abortion and after delivery. RPOC was suspected in 65 cases (28.8%) following vaginal or cesarean delivery and in 161 cases (71.2%) after abortion. Table 1 presents the baseline characteristics of the groups. For all the women, the RPOC was suspected by ultrasound examinations, and hysteroscopy was performed.

**Table 1:** Baseline characteristics of the study population with RPOC: comparison between post-abortion and post-delivery cases. *t-test. ^#^Mann-Whitney. ^**^Fisher. ^##^Chi-square. Statistical value test: t-test, t-value; Mann-Whitney, Mann-Whitney U; Fisher and chi-square, chi-square value. The data are presented as number (%), mean ± standard deviation, or median (range) ART, assisted reproductive technology; RPOC, retained products of conception

Characteristics (*n* = 226)	After abortion, *n* = 161 (71.2%)	After delivery, *n* = 65 (28.8%)	Statistical value test	*P*-value
Age (years)	33 (17-45)	30 (20-43)	1.787	0.075^*^
Pregnancies	3 (1-12)	2 (1-10)	3639.0	<0.001^#^
Cesarean section	0 (0-3)	0 (0-3)	4959.0	0.376^#^
Previous cesarean section	34(21.1%)	11(16.9%)	0.511	0.582^##^
Parity	2 (0-7)	1 (1-8)	5148.500	0.847^#^
Abortions	1 (0-8)	0 (0-7)	1543.500	<0.001^#^
Scared uterus	28 (17.6%)	11 (16.9%)	8.841	0.019^**^
ART	4 (2.5%)	2 (3.1%)	0.063	1.000^**^
Time from the evacuation to the diagnosis (days)	59 (22.25)	77.97 (45.26)	4005.500	0.006^#^
	56 (4-158)	61 (20-247)		
Asymptomatic	81 (50.6%)	21 (32.3%)	6.258	0.018^##^
Bleeding	67 (41.9%)	41 (63.1%)	8.324	0.005^##^
Amenorrhea	1 (0.6%)	0	0.408	1.000^**^
Fever	2 (1.3%)	0	0.820	1.000^**^
Pain	10 (6.3%)	9 (13.8%)	3.450	0.070^##^
Suspected residual by hysteroscopy	143 (88.8%)	52 (80.0%)	3.044	0.090^**^ 0.066 1-sided
Pathological residual	132 (81.9%)	47 (72.3%)	2.634	0.077^##^
Size of residual on hysteroscopy (cm)	1.96 (±0.82)	2.00 (±1.14)	2349.0	0.915^#^
Complete residual extraction	137 (97.2%)	46 (90.2%)	4.069	0.058^**^
Complications	5 (3.1%)	8 (12.4%)	8.834	0.011^**^

No statistically significant differences were observed between the groups regarding age, parity, or the prevalence of a scarred uterus and the use of assisted reproductive technology (ART). Among women with RPOC after abortion compared to those after delivery, the median (range) gravidity was higher [3 (1-12) vs. 2 (1-10), *P* < 0.001], as was the median (range) number of previous abortions [1 (0-8) vs. 0 (0-7), *P* < 0.001]. In the post-abortion group compared to the post-delivery group, the mean time from evacuation to RPOC diagnosis was shorter (59 ± 22.25 vs. 77.97 ± 45.26 minutes; *P* = 0.006). 

A higher proportion of women in the post-abortion group were asymptomatic compared to the post-delivery group (81/161, 50.6%, vs. 21/65, 32.3%; *P* = 0.018]. Bleeding was more common in the post-delivery group (41/65, 63.1%, vs. 67/161, 41.9%; *P* = 0.005]. Pain presented less frequently in the abortion than in the delivery group, but the difference was not statistically significant (10, 6.3%, vs. 9, 13.8%; *P *= 0.070). Other clinical symptoms, like amenorrhea and fever, did not differ significantly between the groups. 

The detection rate of RPOC was higher after abortion than after delivery, though not statistically significant (82.9% vs. 72.3%; *P* = 0.077). In the post-abortion group compared to the post-delivery group, complete residual extraction was more frequent, though not statistically significant (137/141, 97.2%, vs. 46/51, 90.2%; *P* = 0.058). Hysteroscopy-related complications were less common in the post-abortion group (5, 3.1%, vs. 8, 12.4%; *P* = 0.011).

Table 2 presents the sonographic findings according to the source of the RPOC. The groups did not differ significantly in the proportion of women with fluid in the uterus or the mean endometrial thickness (1.67 cm vs. 1.18 cm, *P *= 0.353). Similarly, there was no significant difference in the categorical analysis of endometrial thickness (<10, 10-20, and >20 mm) between the groups (Table 2). However, Doppler flow was more prevalent among women with RPOC following abortion compared to those with RPOC after delivery (70.8% vs. 55.4%, *P *= 0.030).

**Table 2:** Sonographic findings of RPOC: comparison between post-abortion and post-delivery cases. *t-test. ^#^Mann-Whitney. **Fisher. ##Chi-square. Statistical value test: t-test, t-value; Mann-Whitney, Mann-Whitney U; Fisher and chi-square, chi-square value. The data are presented as number (%), mean ± standard deviation, or median (range) ART, assisted reproductive technology; RPOC, retained products of conception

Characteristics (*n *= 226)	After abortion, *n *= 161 (71.2%)	After delivery, *n *= 65 (28.8%)	Statistical value test	*P*-value
Thickness (cm) on transvaginal sonography	1.67 (0.58); 1.6 (0.7-4.5)	1.83 (0.93); 1.7 (0.8-6)	4818.500	0.353^#^
Liquid in the uterus	10 (6.2%)	7 (10.8%)	1.383	0.268^##^
Doppler flow	114 (70.8%)	36 (55.4%)	4.935	0.030^##^
Endometrial thickness				
<10 mm	17 (10.6%)	5 (7.7%)	4674.500	0.128^#^
10-20 mm	112 (69.6%)	41 (63.1%)		
>20 mm	32 (19.9%)	19 (29.2%)		

**Figure 1:**
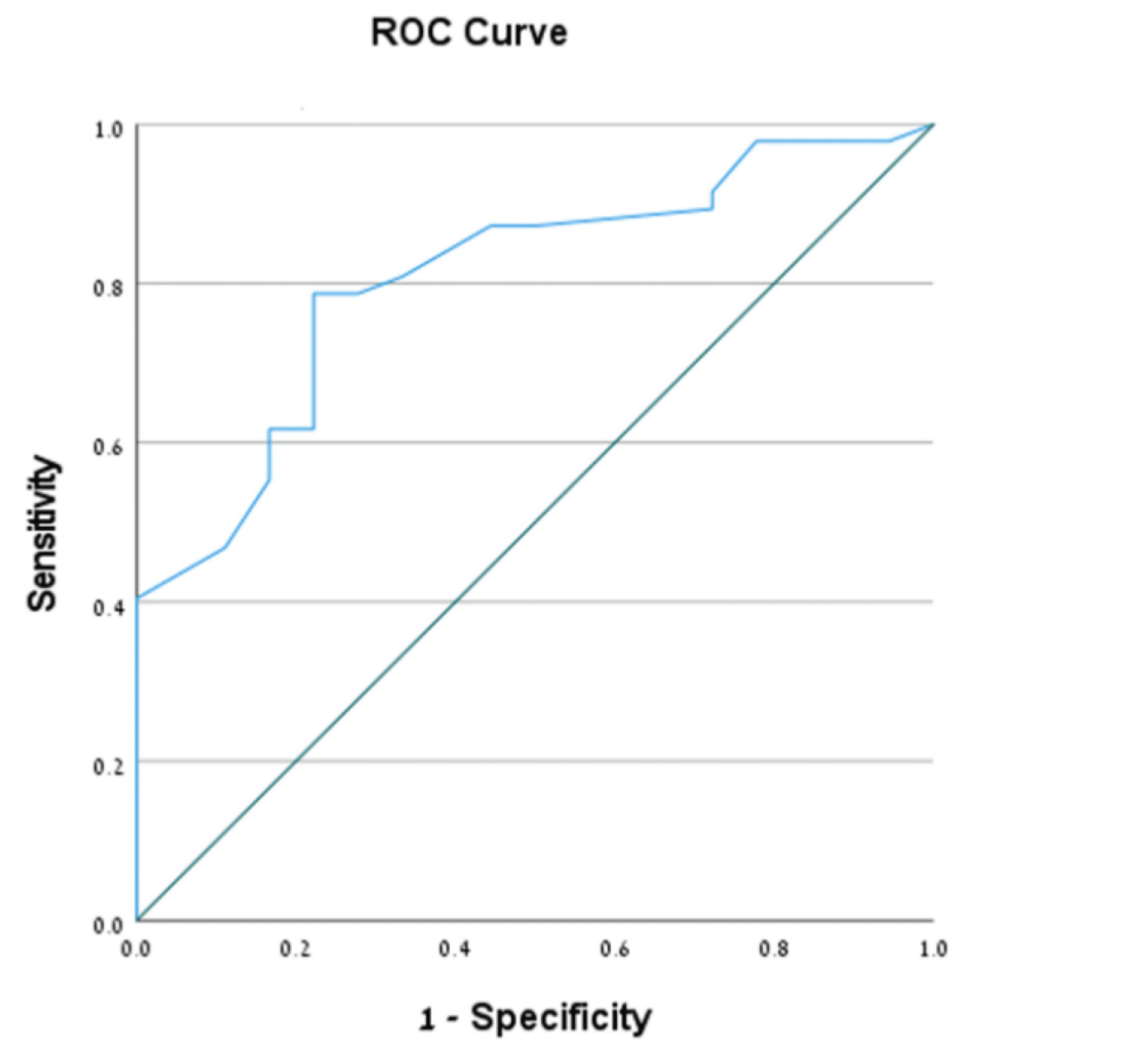
ROC curve analysis evaluating the association between endometrial thickness, as measured by transvaginal sonography, and the detection of retained products of conception following abortion. ROC, receiver operating characteristic

Receiver operating characteristic (ROC) analysis demonstrated that, for the prediction of RPOC following abortion, the area under the curve (AUC) for endometrial thickness was 0.695 (95% confidence interval [CI]: 0.595-0.795, *P* < 0.001) (Figure 1). The optimal cutoff value was 1.55 cm, providing a sensitivity of 60% (95% CI: 51.0%-68.3%), specificity of 72.4% (95% CI: 52.8%-87.3%), and a positive predictive value (PPV) of 90.8% (95% CI: 82.7%-96.0%).

**Figure 2:**
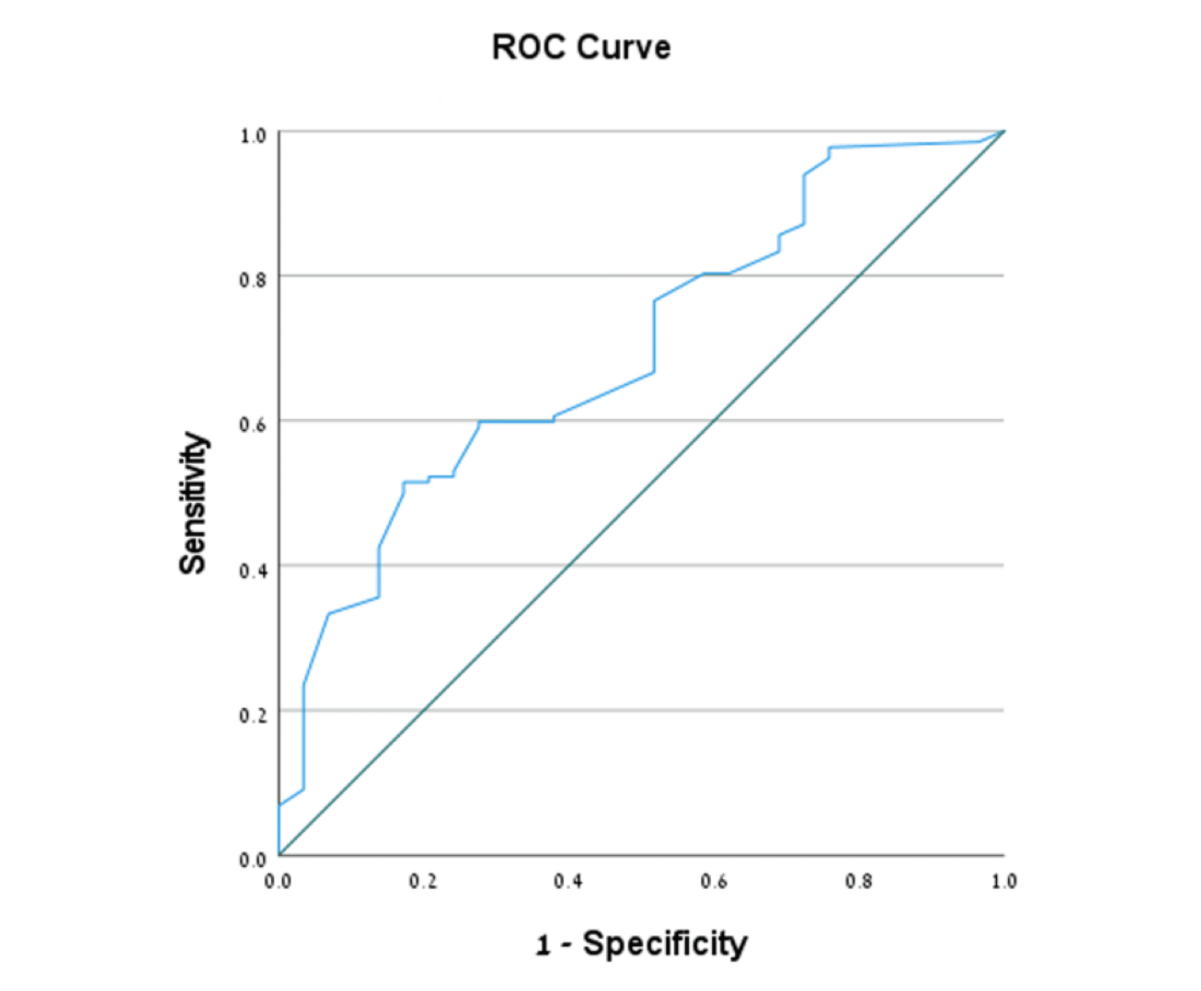
ROC curve analysis evaluating the association between endometrial thickness, as measured by transvaginal sonography, and the detection of retained products of conception following delivery ROC, receiver operating characteristic

In predicting RPOC after delivery, the AUC for endometrial thickness was 0.808 (95% CI: 0.698-0.918, *P* < 0.001). An optimal threshold of 1.49 cm yielded a sensitivity of 78.7% (95% CI: 64.3%-89.3%), specificity of 77.8% (95% CI: 52.4%-93.6%), and a PPV of 90.2% (95% CI: 76.9%-97.3%) (Figure 2).

## Discussion

We identified distinct demographic and obstetric profiles among women with RPOC after delivery versus after abortion. While age, parity, scarred uterus, and ART did not differ significantly between the groups, women with RPOC post-abortion had higher gravidity and a greater history of abortion. Additionally, the mean time to RPOC diagnosis was shorter after abortion than after delivery (59 ± 22.25 vs. 77 ± 45.26 minutes, *P* = 0.006). These intervals have been reported within the range of 55-107 days [11,12].

Notably, about half of post-abortion and one-third post-delivery were asymptomatic. Conversely, vaginal bleeding and abdominal pain were more common post-delivery (41.9% vs. 63.1%, *P*=0.005; 6.3% vs. 13.8%, *P *= 0.070, respectively). The variance in symptoms between groups may be attributed to differences in uterine physiology, evacuation processes, and RPOC size, as well as to variations in surveillance protocols. 

Our results are consistent with previous research indicating that approximately 30% of women diagnosed with RPOC were asymptomatic [5]. Other studies have found that vaginal bleeding was present in only 40%-70% of RPOC cases [12,13]. These high rates of asymptomatic presentations underscore the need for routine evaluation for RPOC - particularly following abortion - even in the absence of overt symptoms. We report marginally better sensitivity in detecting RPOC post-delivery than post-abortion. However, the overall diagnostic performance was comparable between the groups (82.9% vs. 72.3%, *P *= 0.077), consistent with a recent meta-analysis [14]. There was no significant difference in endometrial thickness between the two groups. However, Doppler flow was more prevalent in the post-abortion than the post-delivery group (114, 70.8%, vs. 36, 55.4%, *P *= 0.030). This suggests that Doppler flow may hold greater diagnostic value for women with RPOC following abortion. Although RPOC diagnosis typically involves pelvic ultrasound, often complemented by Doppler flow tests [15,16], the effectiveness of Doppler in triaging women with suspected RPOC remains limited. Sonographic features in women after abortion or delivery showed considerable overlap between those with and without RPOC [17]. In these scenarios, sonography tests have shown low PPVs and a notable false-positive rate (48.5%-71% and 28%-51%, respectively) [11,18]. 

Using ROC analysis, we report an endometrial thickness cutoff for predicting RPOC post-abortion of 1.55 cm and for predicting RPOC post-delivery of 1.49 cm. In the post-abortion analysis, the sensitivity was 60.0%, the specificity was 72.4%, and the PPV was 90.8%. In the post-delivery analysis, these respective values were 78.7%, 77.8%, and 90.2%. Endometrial thickness is the most used diagnostic measure for predicting RPOC, irrespective of its origin [19]. We determined that both cutoff values represent moderate to good diagnostic accuracy, while the sensitivity and specificity were slightly better in the post-delivery group. These findings underscore the importance of endometrial thickness assessment in RPOC diagnosis, regardless of the antecedent event. 

The distinct differences in the clinical presentation and diagnosis of RPOC following delivery versus abortion underscore the need for context-specific diagnostic criteria. These findings expand upon our previous study comparing symptomatic and asymptomatic RPOC, revealing significant diagnostic disparities and emphasizing the importance of routine sonographic evaluation in asymptomatic women with risk factors. Moreover, our data suggest that incorporating Doppler flow into the sonographic assessment markedly enhances diagnostic accuracy, particularly among symptomatic women [20].

The strengths of our study include a single-institute cohort managed with standardized protocols and confirmation of RPOC through hysteroscopy and pathologic assessment. To our knowledge, this is the first analysis to compare RPOC characteristics between women post-delivery and post-abortion directly. However, limitations, including the retrospective design and potential selection bias, warrant caution in interpreting our findings. Additionally, the heterogeneous nature of the RPOC occurrences after various abortion and delivery modalities prompts the need for prospective studies with larger sample sizes to validate our results.

## Conclusions

Our study sheds light on the nuanced differences in clinical presentation, diagnostic accuracy, and sonographic features of RPOC post-delivery versus post-abortion. Regular sonographic evaluation for RPOC detection is recommended, especially for high-risk women, even when symptoms are absent, particularly following abortion. Understanding the distinctions delineated is crucial for optimizing diagnostic strategies and informing tailored management approaches for women at risk of RPOC. Prospective investigations are warranted to validate our findings and elucidate additional factors that influence RPOC diagnosis and management.
